# Neuroprotective Potential of Phytocompounds in the Treatment of Dementia: The State of Knowledge from the Scopolamine-Induced Animal Model of Alzheimer’s Disease

**DOI:** 10.3390/cimb47080635

**Published:** 2025-08-08

**Authors:** Joanna Szala-Rycaj, Mirosław Zagaja, Aleksandra Szewczyk, Jolanta Polak, Marta Andres-Mach

**Affiliations:** 1 Department of Experimental Pharmacology, Institute of Rural Health, Jaczewskiego 2, 20-090 Lublin, Poland; szala-rycaj.joanna@imw.lublin.pl (J.S.-R.); szewczyk.aleksandra@imw.lublin.pl (A.S.); andres.marta@imw.lublin.pl (M.A.-M.); 2 Department of Biochemistry and Biotechnology, Institute of Biological Sciences, Maria Curie-Skłodowska University, Akademicka 19, 20-033 Lublin, Poland; jolanta.polak@mail.umcs.pl

**Keywords:** dementia, Alzheimer’s disease, scopolamine, polyphenols, alkaloids, terpenoids, flavonoids

## Abstract

Dementia is a broad category of neurodegenerative pathologies characterized by a progressive decline in two or more cognitive domains, including memory, language, executive and visuospatial functions, personality, and behavior, resulting in the loss of the ability to perform instrumental and/or basic daily activities. One of the most common types of dementia is Alzheimer’s disease. Current approved treatments for Alzheimer’s disease are mainly limited to alleviating cognitive, behavioral, and psychological deficits. To date, four drugs belonging to two families have been approved for the treatment of Alzheimer’s disease: acetylcholinesterase inhibitors (donepezil, galantamine, rivastigmine) and antiglutamatergic drugs (memantine). Drugs delay the progression of the disease, but they cause a number of side effects. Many scientific studies have focused on finding natural products with potential neuroprotective properties and no or minimal cytotoxicity that can support current drug therapy. The main objective of this review is to analyze and describe the neuroprotective potential of selected groups of natural substances (polyphenols, alkaloids, terpenoids) in one of the commonly performed in vivo studies, the scopolamine-induced animal model of Alzheimer’s disease. The article is a review of literature reports from the last 5 years, and the information collected indicates that the neuroprotective activity of natural compounds may prove to be a potential alternative or add-on for Alzheimer’s disease therapy.

## 1. Introduction

### 1.1. Dementia—Characteristics, Incidence

Dementia, or dementia syndrome, is more than just a “memory disorder.” It can manifest itself as a growing impairment in attention, speech, cognitive abilities, and orientation. This disease affects perception and behavior [1]. The prevalence of dementia is predicted to triple in 2050 compared to that in 2019, reaching 150 million cases worldwide [2]. Normal aging can be defined as a natural biological process in older people characterized by relative brain atrophy without major impairment of normal cognitive and motor functions. The aging brain shows a decrease in volume, usually associated with diffuse or focal white matter signal abnormalities. However, there is no clear clinical or pathological boundary between physiological and abnormal brain aging [3]. Most often, dementia in the elderly is caused by progressive neurodegeneration.

### 1.2. Alzheimer’s Disease as a Type of Dementia—Pathophysiology

The most common degenerative dementias in older adults include Lewy body dementia, vascular dementia, frontotemporal degeneration, Parkinson’s disease, and Alzheimer’s disease (AD). The last two diseases mentioned account for 60–80% of all cases of dementia [4]. Based on the currently known risk factors for AD and the processes underlying its pathophysiology, three hypotheses/mechanisms can be distinguished for the development of AD: the cholinergic hypothesis, which relies on the disruption/loss of cholinergic neurotransmission, specifically acetylcholine in the central nervous system (CNS); the formation of neurotoxic amyloid-β, which aggregates into plaques and impairs neuronal communication; and the accumulation of Tau protein, which becomes hyperphosphorylated, damaging neurons [5]. The oldest, most widely known and accepted mechanism is the cholinergic hypothesis. The normal cholinergic system in the brain is known to influence hippocampal neurogenesis and cognitive function by modulating neurogenic mechanisms such as those involving brain-derived neurotrophic factor (BDNF) and cAMP response element binding protein (CREB). Impaired learning and memory functions associated with aging are primarily attributed to cholinergic dysfunction, including impaired acetylcholine (ACh) release and increased acetylcholinesterase (AChE) activity in CNS neurons [6,7].

### 1.3. Treatment Methods for AD

Currently approved treatments for AD includes four drug forms (the AChE inhibitors donepezil, galantamine, and rivastigmine and the antiglutamatergic drug memantine) and are mainly limited to alleviating cognitive, behavioral, and psychological deficits. AChE inhibitors are molecules designed to increase the level of ACh in the brain, which allows for the transmission of information between neurons and plays an important role in memory. In turn, antiglutamatergic drugs are used to regulate glutamate levels by noncompetitive antagonistic action of N-methyl-D-aspartate receptors. These drugs are used to delay the progression of the disease to stabilize, improve, and control behavioral disorders. Although they do not provide a cure, they help maintain independence and improve quality of life for people with AD and their caregivers [8,9,10,11]. Great hopes were placed in two monoclonal antibodies against amyloid β (Aβ; donanemab and lekanemab), but the publication of the final results from clinical trials showed the limited clinical effect of these drugs [12]. Currently, antidementia drugs and disease-modifying therapies have limited efficacy and are primarily intended for the treatment of AD [13]. Given the increasing prevalence of dementia and the relative inadequacy of currently available pharmacological treatments, there is a need to develop and implement new therapies [14].

**Figure 1:**
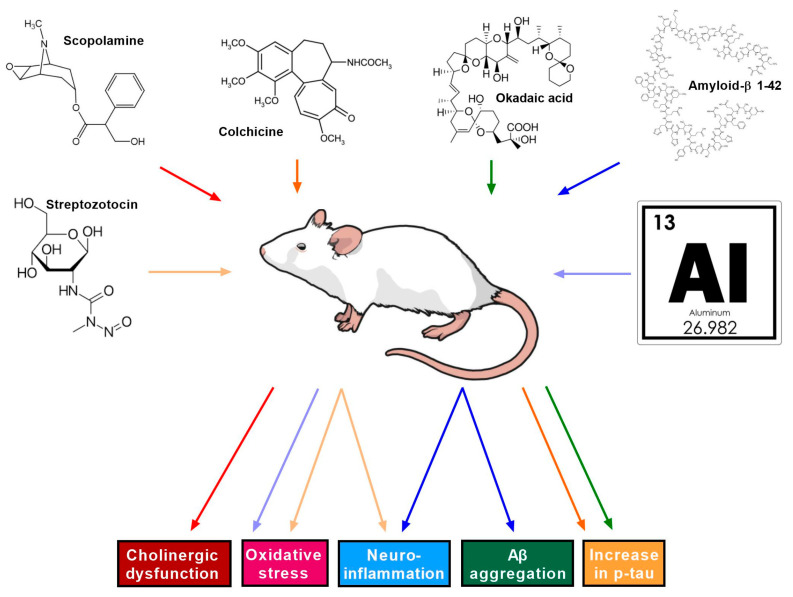
Chemically induced animal models of Alzheimer’s disease. AD induced by administration of streptozocin, scopolamine, colchicine, okadaic acid, amyloid β, and aluminum leading to cholinergic dysfunction, oxidative stress, neuroinflammation, Aβ accumulation, and p-tau increase.

The first stage of developing new therapies is to conduct a series of detailed preclinical in vitro and in vivo studies. After confirming their efficacy and safety in preclinical studies, new drug candidates can be prepared for clinical trials. In chemical animal models of Alzheimer’s disease, depending on the mechanism of action and neurotoxic effect of the selected substance, we can establish different types of AD models (Figure 1) [15].

Animal models based on cholinergic neurodegeneration seems to be a good choice for first-stage studies used for screening antidementia drugs. So far, the only model using this mechanism is a scopolamine (SCO)-induced AD model. SCO is widely used in basic in vivo research studies to induce an impairment in cognitive functions and memory that is very similar to AD in terms of the main characteristics, which are cholinergic dysfunction and the accumulation of amyloid-β plates. Additionally, SCO induces changes such as impaired antioxidant defense systems, increased oxidative stress, mitochondrial dysfunction, apoptosis, and neuroinflammation, which mimic those that occur in AD patients. Although the SCO model has some limitations, it is one of the best and easiest in vivo models for use in research on AD [16,17].

Bearing in mind the fact that Alzheimer’s disease belongs to the group of incurable neurodegenerative disorders caused by the loss/atrophy/degeneration of neurons and available treatments either delay the progression of the disease or cause side effects in patients, it is extremely important to search for a substance that will act neuroprotectively without causing adverse reactions in patients [18]. In recent years, natural compounds have gained interest from researchers due to their wide range of biological activities, including strong neuroprotective effects and no or minimal cytotoxicity [19]. The main objective of this review is to analyze and present the latest research data indicating the positive effect of therapy with three groups of phytochemical—polyphenols, alkaloids, and terpenoids—in the treatment of SCO-induced AD in animals.

## 2. Materials and Methods

The name of the natural chemicals and their connection to the SCO-induced AD model were among the desired reporting items, and a methodical search technique guided the writing of this review study. We searched the following databases: PubMed^®^ (https://pubmed.gov/; US National Library of Medicine, Bethesda, MD, USA, accessed on 12 March 2025), Web of Science^®^ (https://www.webofknowledge.com, Thomson Reuters, Philadelphia, PA, USA, accessed on 25 March 2025), and SciVerse Scopus^®^ (https://www.scopus.com, Elsevier Properties S.A., Amsterdam, The Netherlands, accessed on 7 April 2025). Three key concepts that were pertinent to the three research questions—the disease condition (dementia or Alzheimer’s disease), the experimental model (SCO-induced), and the intervention (natural compounds, including phytochemicals and particular classes like polyphenols, alkaloids, terpenoids, and flavonoids)—were identified in order to develop the search strategy. The search strings were created using Boolean operators (AND, OR). An example of a Boolean string used to prepare this review study is as follows: “Alzheimer Disease” OR “Alzheimer’s disease” OR “dementia”) AND “scopolamine/SCO” OR “SCO-induced” AND “natural compound” OR “phytochemical” OR “plant extract” OR “polyphenol*” OR “alkaloid*” OR “terpenoid” OR “flavonoid*” AND “in vivo” OR “animal model*” OR “rodent”.

Only peer-reviewed, original research papers and the 9 most significant review articles written in English were taken into account. Studies that used animal models (such as rats) in vivo, examined the impact of natural chemicals on AD pathogenesis or SCO-induced cognitive dysfunction, and reported behavioral, biochemical, or histological outcomes pertinent to AD were also considered for inclusion. In vitro research, editorials, conference abstracts, and studies without SCO-induced models were excluded.

**Figure 2:**
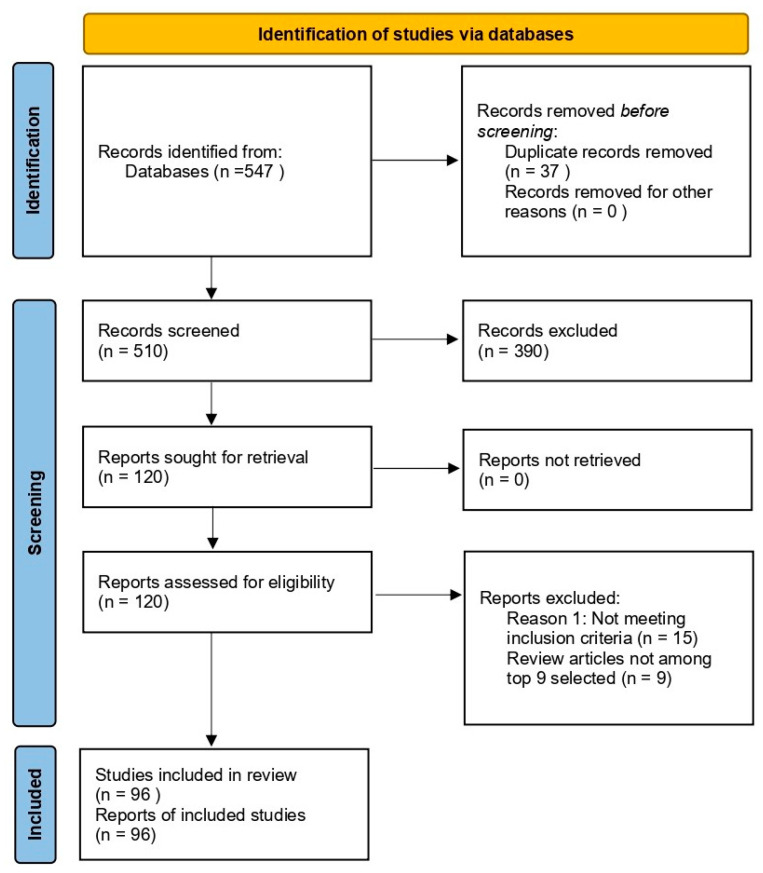
PRISMA-based flow diagram of study selection for systematic review. Summary of the identification, screening, and inclusion process for studies selected in this systematic review.

The search items included dementia; Alzheimer’s disease; SCO-induced model of AD; natural substances classified by type as polyphenols, alkaloids, terpenoids, or flavonoids; and in vivo studies of phytochemicals in the treatment of SCO-induced AD. A total of 547 articles were identified in the initial phase. Finally, after content analysis, only 96 matching articles were selected. PRISMA guidelines were used for reporting articles (Figure 2).

## 3. Scopolamine-Induced Animal Model of AD

**Figure 3:**
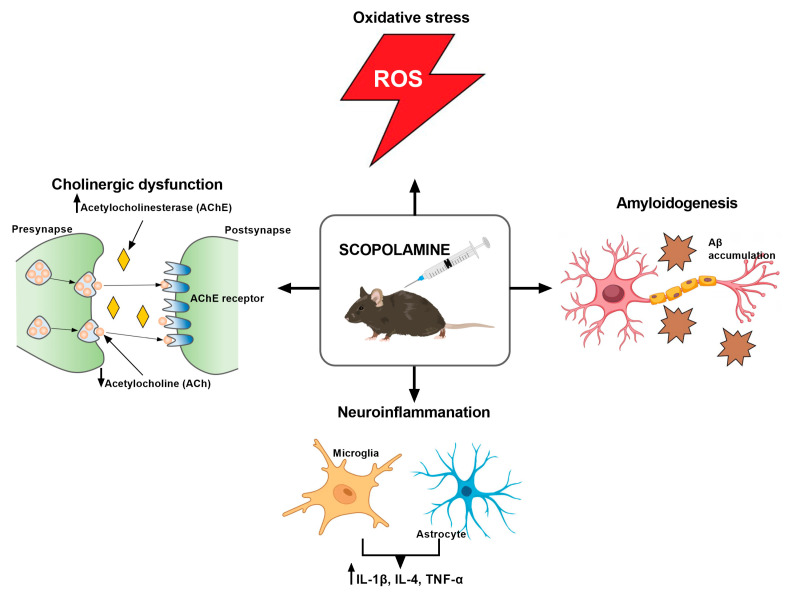
Scopolamine—mechanisms of action.

SCO is a tropane alkaloid obtained from *Hyoscyamus niger* and has a wide range of applications in medicine [20]. SCO a non-selective muscarinic receptor antagonist that causes cholinergic dysfunction by disrupting the regulation of AChE, one of the most important neurotransmitters in memory processing. Due its high lipophilicity, SCO easily crosses the blood–brain barrier (BBB) and acts in the CNS, leading to the appearance of problems with spatial memory and electrophysiological changes that resemble the symptoms of AD [16,20,21,22]. Additionally, SCO advances the cleavage pathway of alternative amyloid precursors, which causes the development of amyloid β plaques (Figure 3) [23].

Apart from SCO, atropine is another tropane alkaloid that has anticholinergic properties. Both substances can penetrate the blood–brain barrier and impact the central nervous system. Anxiety, delirium, and hallucinations are possible adverse effects of both atropine and scopolamine. The primary distinction between these substances is that, when given in therapeutic dosages, atropine has a mild effect on the central nervous system (CNS), whereas SCO, when given in similar dosages, has a stronger effect and is more likely to induce sedation and amnesia than atropine, which can result in cognitive impairment and disorientation [24,25].

Most in vivo studies have shown that SCO can cause the atrophy of brain neurons, as well as induce the accumulation of Aβ in the brain by increasing the activity of tau kinase, which is involved in the pathogenesis of AD [26,27,28]. This compound is also able to disrupt the functioning of cholinergic circuits by reducing the level of ACh and increasing the activity of AChE, which impairs cognitive functions in the rodents, similarly to the neurodegenerative symptoms in human AD [26,29,30,31,32,33,34,35]. Other preclinical studies on mice [36,37] and rats [38,39] indicated memory impairment associated with oxidative stress and impaired antioxidant defense mechanisms caused by the accumulation of malondialdehyde (MDA), a characteristic feature of AD. Moreover, SCO has also been shown to have a proapoptotic effect on hippocampal neurons, leading to abnormal apoptosis, which is also related to neurodegenerative diseases (NDs) [40,41]. SCO also shows a pro-inflammatory effect, similar to inflammation in AD, as evidenced by the increase in the levels of IL-1β, IL-4, and TNF-α in the brain after its administration (Figure 3) [42]. Finally, SCO adversely affects brain functions by reducing the level of BDNF [35,43], a factor that plays a key role in neuroprotection and neurogenesis [44]. The model’s limitations are highlighted by Klinkenberg and Blokland [45] and include the possibility of negative outcomes, including changes in pupil width, smooth muscle dysfunction, and salivary secretion. Moreover, SCO does not contribute to the disorder’s progression and has a transient effect. This model continues to be one of the most widely utilized in preclinical research in spite of these drawbacks.

SCO lowers ACh levels, increases AChE activity, induces oxidative stress by accumulating MDA, leads to Aβ accumulation, and has a pro-inflammatory effect by increasing IL-1β, IL-4, and TNF-α levels.

## 4. Phytocompounds in AD Treatment: Findings from the SCO-Induced Model of AD

The scientific community is facing a difficult challenge of finding reliable natural compounds with potential for treating neurodegenerative disorders, including AD. Considering that AD is a multifactorial disease, natural compounds offer a multi-target approach (they exhibit neuroprotective, antioxidant, anti-inflammatory, anti-apoptotic effects, etc.) compared to drugs that often act on a single molecular target. A wide range of natural compounds such as polyphenols, alkaloids, terpenes, and terpenoids have therapeutic potential in AD [46]. Below, we describe the latest studies using these three groups of compounds in an animal model of SCO-induced dementia (Table 1).

### 4.1. Polyphenols

Polyphenols are characterized by strong antioxidant properties but also have a range of biological activities, including neuroprotective effects, important in the treatment of ND [47]. In terms of the structure of the basic carbon skeleton, polyphenolic compounds are divided into phenolic acids and flavonoids [48]. Based on their chemical composition, polyphenols are grouped into families and subfamilies, such as flavonoids (including flavonols, flavan-3-ols, anthocyanidins, flavones, flavanones, isoflavones, and chalcones), phenolic acids, stilbenes, lignans, and saponins [49]. The fact that polyphenols cross the BBB [50] makes them excellent candidates for the adjunctive treatment of ND due to their neuroprotective and neuromodulatory properties, and in recent years, many studies have been conducted confirming their beneficial neuroprotective effects [51,52,53,54].

Polyphenols, due to their biological properties such as anti-inflammatory, immunomodulatory, antioxidant, cardiovascular protective, and anticancer effects, play an important role in many diseases, including neurodegenerative, neoplastic, metabolic, and circulatory disorders [55]. A study conducted by Zeng et al. [56] showed that the neuroprotective effect of flavonoids in AD is associated with the mediation of glycogen synthase kinase 3β (GSK3β) and cyclin-dependent kinase 5 (CDK5). Additionally, many scientific studies indicate a relationship between the neuroprotective effects of polyphenols and the modulation of the composition of the intestinal microbiota, as well as the neurotransmitters and neuropeptides (BDNF, catecholamines, serotonin, dopamine, GABA, and histamine) they produce [57,58]. Polyphenols have also been shown to regulate BDNF and nerve growth factor (NGF) levels and CREB protein, which are important for neurogenesis, neuronal survival, learning, and memory in the brain and therefore for cognitive performance [59,60]. Another mechanism of action of polyphenols is the antioxidant effect by inhibiting nitric oxide (NO) and prostaglandin E2 (PGE2) production, which eliminates the formation of reactive oxygen species (ROS) in the brain, contributing to the neuroprotective effect [61]. The antioxidant effect of polyphenols also affects the degradation of amyloid precursor proteins, thus protecting against the deposition of AB plaques [62]. It has also been shown that polyphenols inhibit AChE, which can prevent dementia [63]. In addition, polyphenols, thanks to their anti-inflammatory properties, block the release of the unfavorable pro-inflammatory cytokines [64].

In recent years, polyphenols have attracted much interest in the area of in vivo studies using the SCO model of AD. Liu et al. [65] evaluated the effect of 4 weeks of supplementation with resveratrol oligomers from *Paeonia suffruticosa* seed husks (150 mg/kg and 600 mg/kg) in a mouse (C57BL/6J) model of SCO (1.5 mg/kg)-induced AD. Behavioral tests (Morris water maze MWM test, novel object recognition NOR test) showed an improvement in cognitive function. In turn, the measurement of several selected biochemical markers levels showed an increase in AChE, cholineacetyltransferase, ACh, superoxide dismutase (SOD), catalase (CAT), and glutathione (GSH) in the mouse brain and interleukin-4 (IL-4) in the serum, as well as a decrease in interleukin-1β (IL-1β), interleukin-6 (IL-6), and tumor necrosis factor alpha (TNF-α) in the serum of SCO mice, which clearly confirms the anti-inflammatory effects of resveratrol. Another flavonoid apigenin administered at dose of 10 mg/kg and 20 mg/kg for 14 days significantly alleviated cognitive deficits caused by the injection of SCO (1.5 mg/kg) in T-maze, NOR, and MWM tests in male ICR mice. The biochemical analysis indicated an increase in BDNF and tropomyosin receptor kinase B (TrkB) expression after apigenin administration. Additionally, SCO-induced lipid peroxidation in brains and the expression of apoptosis factors Bax/Bcl-2, capase-3, and poly(ADP-ribose) polymerase (PARP) were significantly reduced. Such results may suggest that this flavonoid regulates the amyloidogenic pathway and promotes Aβ degradation [66].

In turn, Olayinka et al. [67] evaluated the anti-inflammatory and neuroprotective effects of quercetin (12.5 mg and 25 mg/kg) in a mouse SCO (3 mg/kg)-induced dementia model. Quercetin treatment alone and in combination with SCO for 7 consecutive days significantly reversed SCO-induced memory deficits in the NOR test. In addition, an ELISA test showed that the elevated levels of TNF-α and IL-6 were reduced by quercetin, whereas TUNEL staining indicated that quercetin attenuated SCO-induced cell degeneration and death in hippocampal subregions and the prefrontal cortex, confirming that quercetin could become a potential therapeutic agent for the treatment of AD-like conditions. Similarly, an improvement in memory in the elevated plus maze (EPM) and NOR tests after quercetin at a dose of 25 mg/kg in Wistar rats was shown by Safarzadeh et al. [68]. These results were probably related to the prevention of quercetin from cleaving amyloid precursor protein (APP) to generate Aβ and the occurrence of cognitive dysfunction. Another study by Kim et al. [69] evaluated the impact of flavonoid epigallocatechin gallate (EGCG) on cognitive disturbances in a rat SCO-induced model of AD. Sprague–Dawley rats were pre-supplemented with EGCG (5 mg/kg) for 19 days, and then EGCG and SCO (1 mg/kg) were administered 60 and 30 min before the Y-maze, passive avoidance (PA), and MWM tests for the next 9 days. In addition, the measurement of AChE activity, SOD, MDA levels was performed. EGCG was subsequently shown to improve short- and long-term memory during tests performed on rats with memory deficit. Moreover, EGCG decreased AChE activity and MDA levels but increased SOD activity in the hippocampus, suggesting its beneficial effect on improving cognitive functions in the selected model of AD.

### 4.2. Alkaloids

Another group of natural substances that are of great interest to researchers in the context of neuroprotection in the SCO model of AD are alkaloids. They are mainly produced by poppy plants (*Papaveraceae*), legumes (*Fabaceae*), buttercup plants (*Ranunculaceae*), and nightshades (*Solanaceae*) and most often collected in leaves, fruits, and seeds, as well as in flowers, roots, tubers, and bark. The content of alkaloids ranges from trace amounts to 10% and largely depends on the development period of the plant, season, region, or climate [70]. Due to their high and varied biological activity (e.g., reviving/stimulating, hypotensive, diuretic, antiarrhythmic, antiasthmatic, antitussive, anticancer, or antirheumatic), many of them are successfully used in medicine [71].

One of the modes of action of alkaloids (i.e., caffeine) is their anti-inflammatory efficacy by inhibiting NF-κB and the NLRP3 and NLRC4 inflammasomes, thus reducing the production of the inflammatory cytokine IL-1β [72]. In turn, another compound from the alkaloid group, harman, blocks the formation of ROS by inhibiting myeloperoxidase (MPO), which consequently reduces the formation of IL-1α and TNF-α [73]. Trigonelline, a pyridine alkaloid, has shown neuroprotective effects in in vivo models of Alzheimer’s disease [74] by suppressing inflammation through a reduction in TNF-α, IL-1β, and IL-6 expression [75].

Botton et al. [76] assessed the effect of preventive administration of caffeine (10 mg/kg) on recognition memory in a SCO (2 mg/kg)-induced AD model in adult male CF1 mice. They showed that caffeine pretreatment prevented SCO-induced disruption of short-term and long-term memory in a NOR test. In turn, Callahan and coworkers [77] evaluated the effects of nicotine, cotinine, and anatabine in the Y-maze test on Swiss mice and in the NOR test on Wistar rats in the SCO (0.5 mg/kg)-induced memory deficit model (the doses of the substances were selected appropriately for the animals, Table 1). Each of these compounds significantly suppressed the memory deficit in a dose-dependent manner in the Y-maze test in mice. Nicotine (0.25 and 0.5 mg/kg) and anatabine (0.5 mg/kg and 1 mg/kg) showed the strongest effect and completely suppressed the effect of SCO, whereas cotinine worked beneficially at doses of 0.5 and 1 mg/kg. However, the behavioral NOR test on rats showed that only nicotine reversed memory deficits in the selected AD model. Although these three alkaloids come from the same group and bind to nicotinic ACh receptors, nicotine turned out to have the strongest effect.

Considering the fact that SCO increases the level of Aβ fragment 1-42 (Aβ1-42) and superoxide anions (O_2_^• -^), which are important in AD, Joseph et al. [78] evaluated the neuroprotective effect of a 3-week treatment with apocynin (APO 16 mg/kg) and galantamine (GAL 1 mg/kg) on Wistar rats with SCO (2 mg/kg)-induced AD. Cognitive functions were assessed using the PA test, and the obtained results showed significant protection against memory loss in rats treated with APO and GAL. In addition, quantitative analysis of Aβ1-42 levels by the ELISA method revealed a reduction in Aβ1-42 levels in the APO and GAL SCO rats compared to those in the SCO control group. Several years earlier, Busquet et al. [79] became interested in GAL and decided to investigate whether the combination of inactive-dose GAL (0.1 mg/kg) and memantine (0.5 mg/kg) would show an effect on the SCO (1 mg/kg)-induced AD model in male CD1 mice. Spatial memory assessed using the T-maze proved that the combination of both compounds reversed the deleterious effect of SCO. In turn, episodic memory was assessed in the NOR test, where similar results were obtained, as the treatment induced a statistically detectable effect of memory improvement.

Dang et al. [80] evaluated the effect of the 22 days of pretreatment with total alkaloids (TA), including huperzine A (HupA) from the *Huperzia serrata* plant, versus pure HupA on cognitive impairments in a mouse model of SCO (1 or 3 mg/kg)-induced AD. The antioxidant activity of TA and Hup A was measured by 2,2-diphenyl-1-picrylhydrazyl assay. Memory was assessed using Y-maze and MWM tests. The analysis of AChE activity, SOD, CAT, MDA levels showed better neuroprotective effects (decreased AChE activity and MDA levels, increased antioxidant enzyme activity in the hippocampus and cerebral cortex) for TA, which may indicate the key role of the mixture of alkaloids in neuron protection related to a reduction in AChE and oxidative stress.

### 4.3. Terpenoids

Terpenoids and terpenes are another group of organic chemical compounds that occur naturally in all living organisms, including mostly plants but also animals [81]. These names are often used alternatively, but they are two different groups of plant metabolites. Terpenes are simple hydrocarbons made up of isoprene units, with the general formula (C_5_H_8_)n. They can be classified depending on the number of isoprene units in their structure, which leads to the creation of categories such as monoterpenes or sesquiterpenes. Terpenoids, on the other hand, are derivatives of terpenes that have been modified by introducing additional functional groups in the form of heteroatoms: hemiterpenoids, monoterpenoids, sesquiterpenoids, diterpenoids, sesterterpenoids, and triterpenoids [81].

Due to their structural diversity, terpenoids exhibit a number of pharmacological activities, such as antiviral, antibacterial, antimalarial, anti-inflammatory, neuroprotective, hypoglycemic, and anticancer effects, which makes them the subject of many scientific studies. In addition, these compounds are successfully used in the cosmetic, food, aromatherapy, and pharmaceutical industries [82]. Typical neuroprotective mechanisms of action for terpenoids are based on the inhibition of the enzyme monoamine oxidase (MAO) involved in the metabolism of neurotransmitters (serotonin, dopamine, noradrenaline, tryptamine) associated with oxidative stress [83]. The pharmacological efficacy of terpenoids is also related to the regulation of the GABA system, and its dysfunction, as we know, plays an important role in many neurological diseases [84]. Additionally, terpenoids affect dopamine D1 and D2 receptors, which have become the target of newly developing neuropsychological therapies [85,86]. Several studies have shown that large groups of terpenoids exert anti-inflammatory and antioxidant effects by changing the pathways associated with inflammatory and oxidative stress. In turn, terpenes such as D-limonene, terpinolene, and triterpene glycosides play a key role in reducing TNF-α, IL-1, and IL-6 expression in in vivo models [87].

Magadmi et al. [88] assessed whether a 2-week treatment with astaxanthin AST (100 mg/kg), a derivative of carotenes belonging to the tetraterpenes group, would have a neuroprotective effect on C57BL/6J mice in an SCO (2 mg/kg)-induced AD model. In addition, the alkaloid GAL (3 mg/kg) alone and in combination with AST was used in the studies. The behavioral Y-maze test was performed to evaluate cognitive functions in mice, while the ELISA tests were used to estimate the content of AChE and brain biomarkers (MDA, SOD enzyme activity, TNF-α, NO, IL-6, Akt1, and pAkt). Finally, immunohistochemical staining of the hippocampus was performed using an anti-GFAP antibody. The obtained results indicated an improvement in cognitive functions after AST supplementation, and the combination of AST+GAL showed an even stronger effect. Increased AChE activity in the brain after SCO treatment was significantly decreased in both AST and AST+GAL groups compared to the SCO control group. Analysis of the results from the ELISA test showed a reduction in oxidative stress and proinflammatory cytokines, which may be the main reason for the improvement in memory by AST. Lv et al. [89] evaluated the effect of 25-day ginsenoside Rh2 (12.5 and 25 mg/kg) treatment on a model of SCO (0.75 mg/kg)-induced AD in male ICR mice. The beneficial efficacy of Rh2 on memory was proven in the object location recognition and MWM tests. Moreover, Rh2 significantly increased the phosphorylation of the extracellular signal-regulated kinase (ERK) pathway and suppressed oxidative stress in the hippocampus, which indicates the neuroprotective effect of this compound by modulating cholinergic transmission, inhibiting oxidative stress, and activating the ERK-CREB-BDNF signaling pathway.

In turn, a study by Wang et al. [90] aimed to investigate the effect of 2 weeks of administration of ginsenoside from *Panax ginseng* stems and leaves (75 and 150 mg/kg) on memory impairment induced by SCO (3 mg/kg) in ICR mice. The treatment alleviated memory impairment by improving the cholinergic function of AChE and reducing oxidative stress (MDA, GSH, and NO). In addition, ginsenoside increased the expression of BDNF and TrkB. The results showed that the ginsenoside treatment, through the regulation of the PI3K/AKT pathway, inhibited neuroinflammation, which in turn led to an improvement in cognitive functioning. Another study on ginsenoside Re performed by Li et al. [91] tested whether this compound (10 mg/kg and 20 mg/kg administered for 2 weeks) had a protective effect against SCO (3 mg/kg)-induced AD in male ICR mice. The MWM test indicated a significant improvement in learning and memory compared to those in the SCO group. TUNEL and DAPI staining showed a reduction in neuronal damage after ginsenoside Re treatment. Additionally, Western blotting analysis of apoptosis-related proteins showed increased expression of antiapoptotic proteins Bcl-xL and Bcl-2 and decreased levels of proapoptotic proteins Bax, Bad, cytochrome c, and Bak compared to those the SCO control mice. Finally, it was shown that ginsenoside Re could activate the PI3K/AKT signaling pathway, further inhibiting oxidative stress and apoptosis, which exerted a protective effect on mice with SCO-induced AD.

Kruk-Słomka et al. [92] evaluated for the first time the natural cannabinoid compound cannabidiol (CBD, 1 mg/kg), the AChE inhibitor rivastigmine (RG, 0.5, mg/kg), and the combination of CBD (1 mg/kg) + RG (0.5 mg/kg) on the impairment of memory acquisition, consolidation, and retrieval induced by SCO (0.1, 0.3, 1 mg/kg) in Swiss mice. The PA test showed that the administration of both CBD (1 mg/kg) and RG (0.5 mg/kg) significantly alleviated cognitive impairment. In turn, the combined therapy exerted even better effects than the use of these compounds separately, which clearly confirmed its beneficial therapeutic efficacy. Earlier studies by Min et al. [93] using another cannabinoid, N-palmitoyl-5-hydroxytryptamine (Pal-5HT, 0.5 and 1 mg/kg), already indicated a positive effect of this compound on learning and memory deficits induced by SCO (1 or 2 mg/kg) in mice in the MWM and PA tests. In addition, Pal-5HT regulated cholinergic function by inhibiting the increase in AChE activity and the decrease in choline acetyltransferase (ChAT) activity, as well as suppressing oxidative stress and restoring p-CREB and BDNF expression in the hippocampus, which confirms that this compound is certainly worth further advanced research to assess its properties as a drug candidate for preventing neurodegeneration.

**Table 1:** Summary of preclinical in vivo studies on selected phytocompounds in SCO-induced AD models. No side effects were reported in the studies.

Animals Sex	Size of the Studied Population	Substances	Dose and Treatment Schedule	Main Outcomes	References
C57BL/6J Male	Control mice = 15 Treated mice = 60	Resveratrol	150 and 600 mg/kg for 4 weeks orally (p.o.), SCO (1.5 mg/kg; single injection; intraperitoneally (i.p.)	(1) Improved cognitive function (2) Increase in AChE, ChAT, ACh, SOD, CAT, and GSH and decrease in IL-1β, IL-6, and TNF-α	[65]
ICR mice Male	Control mice = 10 Treated mice = 40	Apigenin	10 and 20 mg/kg for 14 days (p.o.), SCO (1.5 mg/kg for 7 days; i.p.)	(1) Improved memory in the T-maze, NOR, and MWM tests (2) Lipid peroxidation in brains and reduced expression of apoptosis factors: Bax/Bcl-2, capase-3, and PARP	[66]
Swiss albino mice Male	Control mice = 7 Treated mice = 42	Quercetin	12.5 and 25 mg/kg for 7 days (p.o.), SCO (3 mg/kg for 7 days; i.p.)	(1) Improved cognitive function (2) Elevated levels of TNF-α and IL-6 reduced by quercetin (3) Attenuation of cell degeneration and death in hippocampal subregions and prefrontal cortex	[67]
Wistar rats Male	Control rats = 7 Treated rats = 49	Quercetin	25 mg/kg for 30 days (p.o.), SCO (1 mg/kg for 9 days; i.p.)	(1) Improved memory in the NOR and EPM test (2) Prevention of quercetin from cleaving APP to generate Aβ	[68]
Sprague–Dawley rats Male	Control rats = 6 Treated rats = 18	Epigallocatechin gallate	5 mg/kg (i.p.) for 19 days, SCO (1 mg/kg for 9 days; i.p.)	(1) Decreased AChE activity and MDA level, increased SOD activity (2) Improved cognitive functions in Y-maze, PA, and MWM tests	[69]
CF1 mice male	Control mice = 14 Treated mice = 335	caffeine	10 mg/kg for 4 days (i.p.), SCO (2 mg/kg; single injection; i.p.)	improved cognitive functions in NOR test	[76]
Swiss albino mice Male	Control mice = 12 Treated mice = 108	Nicotine, cotinine, and anatabine	Nicotine (0.125, 0.25, 0.5 mg/kg; i.p.), cotinine (0.25, 0.5, 1 mg/kg; i.p.), anatabine (0.25, 0.5, 1 mg/kg; i.p., SCO (0.5 mg/kg, single injection; s.c.)	Inhibition of the memory deficit in the Y-maze	[77]
Wistar rats Male	Control rats = 8 Treated rats = 64	Nicotine, cotinine, and anatabine	Nicotine (0.03, 0.1, 0.3 mg/kg; i.p.), cotinine (30, 100 mg/kg; i.p.), anatabine (0.3, 1, 3 mg/kg; i.p.), SCO (0.2 mg/kg, single injection; i.p.)	Nicotine reversed the memory deficits in the NOR test	[77]
Wistar rats Male	Control rats = 11 Treated rats = 33	Apocynin (APO), galantamine (GAL)	APO 16 mg/kg for 3 weeks (p.o.), GAL 1 mg/kg for 3 weeks (i.p.), SCO (2 mg/kg for 6 weeks; i.p.)	(1) Improved cognitive functions in PA test (2) Reduced Aβ1-42 level	[78]
CD1 mice Male	Control mice = 10 Treated mice = 70	Galantamine (GAL), memantine (MEM)	GAL (0.1 mg/kg; s.c.), MEM (0.5 mg/kg; i.p.), SCO (1 mg/kg; i.p.)	(1) Improvement of memory in NOR and T-maze tests (2) Reduced oxidative stress (3) Increased level of neurotransmitters	[79]
Swiss mice Male	Control mice = 10 Treated mice = 60	Total alkaloids (TA) and huperzine A (HupA)	TA 10 and 20 mg/kg, HupA 10 and 20 mg/kg for 22 days (p.o,), SCO (1 or 3 mg/kg, single injection, i.p.)	(1) Neuroprotective effect and decreased AChE activity and MDA levels, increased antioxidant enzyme activity in hippocampus and cerebral cortex for TA (2) Improved cognitive functions in Y-maze and MWM for TA	[80]
C57BL/6J Male	Control mice = 6 Treated mice = 30	Astaxanthin (AST), galantamine (GAL)	AST 100 mg/kg for 14 days (i.p.), GAL 3 mg/kg for 14 days (p.o.), SCO (2 mg/kg for 10 days; i.p.)	(1) Significantly reduced AChE activity (2) Significantly prevented the loss of neurons in the hippocampus (3) Significantly reduced the number of astrocytes (4) Reduced the level of biomarkers of oxidative stress (5) inhibited the development of pro-inflammatory mediators (TNF-α and IL-6)	[88]
ICR mice Male	Control mice = 12 Treated mice = 48	Ginsenoside Rh2	12.5 and 25 mg/kg for 25 days (i.p.) SCO (0.75 mg/kg, single injection; i.p.)	(1) Improved cognitive functions in NOR and MWM tests (2) Increased the phosphorylation of the extracellular signal-regulated kinase (ERK) (3) Suppressed oxidative stress in hippocampus	[89]
ICR mice Male	Control mice = 10 Treated mice = 30	Ginsenoside	75 and 150 mg/kg for 14 days, SCO (3 mg/kg for 7 days)	(1) Alleviated memory impairment (by improving the cholinergic function of AChE and reducing oxidative stress) (2) Increased the expression of BDNF and TrkB	[90]
ICR mice male	Cntrol mice = 10 Treated mice = 30	ginsenoside Re	10 and 20 mg/kg for 14 days (p.o.), SCO (3 mg/kg for 7 days; i.p.)	(1) Improved learning and memory in MWM test (2) Ginsenoside Re treatment alleviated neuronal damage (3) Increased the expression of antiapoptotic proteins Bcl-xL and Bcl-2 while decreasing the level of proapoptotic proteins Bax, Bad, cytochrome c, and Bak (4) Compound could activate the PI3K/AKT signaling pathway, further inhibiting oxidative stress and apoptosis	[91]
Swiss mice Male	Control mice = 12 Treated mice = 84	Cannabidiol	CBD 1mg/kg (i.p.), combination CBD (1 mg/kg; i.p.) + RG (0.5 mg/kg; i.p.), SCO (1 mg/kg, single injection; i.p.)	(1) Administration of CBD and RG alleviated cognitive impairment in PA test (2) The combined therapy gave even better effects	[92]
ICR mice Male	Control mice = 9 Treated mice = 45	N-Palmitoyl-5-hydroxytryptamine	0.5 and 1 mg/kg for 3 days (p.o.), SCO (1 or 2 mg/kg, single injection; i.p.)	(1) Improved cognitive functions in PA and MWM tests (2) Inhibited the increase in AChE activity and the decrease in ChAT activity (3) Suppressed oxidative stress and restored p-CREB and BDNF expression in the hippocampus	[93]

## 5. Limitations and Challenges of SCO-Induced Model in AD Research

As was previously noted, the SCO-induced model of AD is one of the most often used screening/first-line experimental models for researching memory and cognitive impairments in animals. Learning and memory are hampered by the reversible reduction in cholinergic transmission caused by SCO, a non-selective antagonist of muscarinic receptors [94]. Preclinical research into possible cognitive-enhancing drugs and therapies for neurodegenerative diseases such as Alzheimer’s often uses this paradigm. One of the most important advantages of using the SCO model is its speed and simplicity. The model is simple to use and does not require genetic engineering or complex surgical techniques. What is more, SCO needs to be administered only once to rapidly induce cognitive impairment. Another major advantage is the reversibility of the effects. Because SCO has a temporary effect, the same animals can be tested both before and after the intervention. For this reason, cross-sectional studies can be conducted on fewer animals. The standardization of methods and reproducible results are possible due to the ability to precisely dose SCO. This ensures robust experimental control and facilitates comparison of the effects of different test agents. Due to the model’s widespread use in memory research and widely available description in the scientific literature, a sizable body of comparative data can be consulted. Scopolamine’s ability to be used in a variety of traditional behavioral and cognitive tests, including the Morris water maze, Y-maze, NOR test, and passive avoidance test, is undoubtedly a huge benefit. The model is helpful in screening cognitive-enhancing drugs because it partially replicates the cholinergic deficiency seen in Alzheimer’s disease.

Despite the many benefits of using this model for in vivo studies, there are also drawbacks and problems, including the lack of structural neurodegenerative changes. SCO causes functional problems but does not cause nerve cell destruction or progressive neurodegeneration, so it is not a good model for chronic neurodegenerative diseases. Its short working time is another major drawback—after a few hours, scopolamine wears off. This makes it difficult to assess the therapeutic effects of drugs and cognitive changes in the long term. Moreover, this paradigm has no applications to drugs. All muscarinic receptors are affected by the non-selective action of SCO. This can cause additional side effects, such as sedation and motor impairment, which can affect behavioral test results. An additional problem with this paradigm is the lack of disease progression (Table 2).

In cognitive research, the SCO-induced AD model is a useful tool, especially for evaluating new neuroprotective drugs and acetylcholinesterase inhibitors. However, its limitations should be kept in mind, the most important of which are its short duration of action and lack of neurodegenerative character. As a result, the model should be viewed as an adjunct to more complex models of neurodegenerative diseases, not as a replacement for them.

## 6. Future Directions for the SCO-Induced Model in AD Research

As the demand for effective treatments for neurodegenerative disorders like AD increases, the SCO-induced dementia model is becoming more and more significant as a supporting tool for screening and functional research. Despite not reflecting all aspects of AD pathology, such as tau hyperphosphorylation, β-amyloid deposition, and chronic inflammation, it is still a very useful model for quickly and affordably evaluating memory impairment and the potential therapeutic benefits of different substances, particularly those that are naturally occurring.

**Figure 4:**
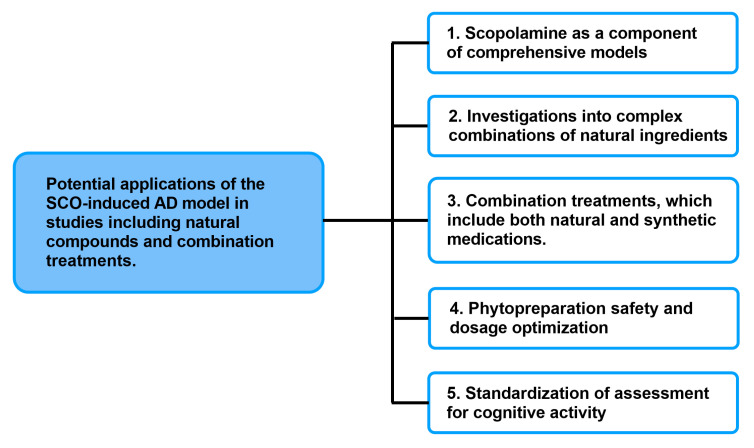
Potential applications of the SCO-induced AD model in studies including natural compounds and combination treatments.

Given the present applications of SCO-induced AD in scientific studies, it can certainly be effectively utilized in investigations involving natural substances and combination therapies (Figure 4). The incorporation of the SCO model into integrated experimental methods, which include both transgenic and toxic models, ought to be one of the primary avenues for future research. In light of the more intricate and protracted pathological alterations seen in AD, this kind of method will provide functional verification of the compounds’ actions. Thus, in preclinical research, the SCO model can serve as a screening tool to help identify the most promising treatment options.

Another important direction is the use of this paradigm in studies of complex plant-based combinations, which are difficult to investigate pharmacokinetically and pharmacodynamically due to their multi-component nature. Unlike studies that focus on individual active chemicals, the SCO model allows for the assessment of the synergistic effect of plant components and their influence on working memory and learning. This could be highly important in the area of complementary and alternative medicine.

Analyzing how synthetic medications interact with natural chemicals is another crucial study angle. The SCO model can be used to evaluate possible pharmacological interactions and side effects of combination medicines, which are becoming more and more popular. This enables an initial evaluation of the efficacy and safety of novel treatment strategies meant to act concurrently on multiple AD pathogenesis pathways.

Determining safe and effective dosages of active ingredients can also be greatly aided by this paradigm. There is little doubt that using a safe and effective dosage is the most important part of therapy. Even though SCO-induced animal models of dementia showed encouraging neuroprotective benefits, the efficacy of phytocompounds are frequently limited by restricted blood–brain barrier (BBB) permeability, low oral absorption or high liver metabolism, and fast conjugation and excretion, which lead to limited systemic bioavailability and uneven therapeutic results. These natural substances may have limited therapeutic windows or dose-dependent toxicity despite occasionally having higher bioavailability [95]. Although various routes of administration of phytocompounds, such as oral administration, inhalation, or topical application to the skin, are mentioned in the literature, Table 1 makes clear that in the case of animal SCO-induced dementia, injections are the main method of administration. Recent studies have looked into enhanced delivery technologies such as liposomes, nanoencapsulation, and phytosome formulations to reduce the limitations and to increase the therapeutic potential of natural drugs [96].

The ability to quickly evaluate the impact on cognitive processes allows for the optimization of dosages for subsequent research in long-term and chronic models. This methodology can also be used to standardize cognitive activity screening tests for natural compounds, which can result in the creation of standardized research procedures for these kinds of drugs.

## 7. Conclusions

The SCO model is a useful, sensitive, and quick research tool that cannot be overstated, even though it is notable to replace more sophisticated neurodegenerative models. From the standpoint of future studies, it might serve as a basis for the creation of novel therapeutic approaches, particularly in the areas of integrated model approaches, natural therapies, and combination therapies in the study of Alzheimer’s disease.

Despite the availability of several drugs that act symptomatically in AD but do not affect the mechanism of disease development, it is important to search for and study natural compounds in AD models, both individually and in combination with existing drugs. It has been shown that compounds such as polyphenols, alkaloids, and terpenes/terpenoids have a strong pharmacological effect and a wide spectrum of action. They penetrate the BBB and have neuroprotective properties, which are extremely important in the treatment of ND. In light of the results of numerous in vivo studies, there is no doubt that due to the prevalence of these compounds in the plant world and their presence in the diet, these compounds are of great importance in the prevention of many societal diseases and may prove effective as adjunctive therapies in the treatment of AD. Thus, polyphenols, alkaloids, and terpenoids/terpenes may be useful in the development of new therapeutic strategies in the prevention and treatment of AD. Although the antidementia effect has been partially verified in preclinical studies, there is a great need for further studies in more complex animal models of AD before they can be included in clinical trials as antidementia drug candidates.

## Figures and Tables

**Table 2:** Advantages and disadvantages of the SCO-induced model of AD.

Advantages	Disadvantages
Quick and easy to use	Does not reflect neurodegeneration
Reversible effect	Short-term effect
Well-controlled dose	Low pharmacological specificity
Well-established in the literature	No progression of disorders
Useful in behavioral tests	Possibility of side effects
Partial similarity to AD	High variability of individual response

## Abbreviations

The following abbreviations are used in this manuscript:

ACh	acetylcholine
AChE	acetylcholinesterase
AD	Alzheimer’s disease
APO	apocynin
APP	amyloid precursor protein
AST	astaxanthin
BBB	Blood–brain barrier
BDNF	brain-derived neurotrophic factor
CAT	catalase
CBD	cannabidiol
CDK5	cyclin-dependent kinase 5
ChAT	choline acetyltransferase
CNS	central nervous system
CREB	cAMP response element binding protein
EGCG	epigallocatechin gallate
EPM	elevated plus maze
ERK	extracellular signal-regulated kinase
GABA	gamma-aminobutyric acid
GAL	galantamine
GSH	glutathione
GSK3β	glycogen synthase kinase 3β
HupA	huperzine A
IL-1β	interleukin-1β
IL-4	interleukin-4
IL-6	interleukin-6
i.p.	intraperitoneally
MAO	monoamine oxidase
MDA	malondialdehyde
MEM	memantine
MPO	myeloperoxidase
MWM	Morris water maze
ND	neurodegenerative disease
NGF	nerve growth factor
NO	nitric oxide
NOR	novel object recognition
PA	passive avoidance	
PGE2	prostaglandin E2
p.o.	orally
ROS	reactive oxygen species
SCO	scopolamine
SOD	superoxide dismutase
TA	total alkaloids
TNF-α	tumor necrosis factor alpha
TrkB	tropomyosin receptor kinase B
